# Risk factors for suicide in Bali: a psychological autopsy study

**DOI:** 10.1186/1471-2458-9-327

**Published:** 2009-09-09

**Authors:** Toshiyuki Kurihara, Motoichiro Kato, Robert Reverger, I Gusti Rai Tirta

**Affiliations:** 1 Department of Psychiatry, Komagino Hospital, Tokyo, Japan; 2 Department of Neuropsychiatry, Keio University School of Medicine, Tokyo, Japan; 3 Department of Psychiatry, University of Udayana, Bali, Indonesia; 4 Bangli Mental Hospital, Bali, Indonesia

## Abstract

**Background:**

The suicide rate in Bali has significantly increased in recent years. However, to date, there have been no case-control studies investigating risk factors for suicide.

**Methods:**

A psychological autopsy study was conducted comparing 60 suicide cases and 120 living controls matched in age, sex, and area of residence.

**Results:**

Multiple logistic regression analysis identified the following risk factors for suicide: at least one diagnosis of axis-I mental disorder (OR: 14.84 CI: 6.12 - 35.94); low level of religious involvement (OR: 7.24 CI: 2.28 - 22.95); and severe interpersonal problems (OR: 3.86 CI: 1.36 - 11.01). Forty-eight (80.0%) of the suicide cases were diagnosed with mental disorders; however, only 16.7% visited a primary care health professional and none received psychiatric treatment during the 1 month prior to death.

**Conclusion:**

Clinical, religious, and psychosocial factors were associated with suicide. These results highlight the significance of early recognition and treatment of mental disorders, religious activities, and interpersonal problem-solving strategies for suicide prevention in Bali.

## Background

Suicide is one of the major causes of death worldwide [1]. In Bali, Suryani et al. [2] demonstrated that the suicide rate in the period following the 2002 Bali bombings was 8.10 for males and 3.68 for females per 100,000 population. Following the bombings, suicide rates increased nearly four-fold in males and three-fold in females [2]. Although the suicide rate is lower than the world average, these results suggest that suicide should be recognized as a major health problem in Bali. Given that this figure is probably underestimated due to underreporting of suicides, the actual impact of suicide on mortality in Bali is most likely much higher. Furthermore, as the Balinese Hindu religion considers suicide to be an offense against the gods that leads to punishment in the next life [3], suicide is a major social concern in Bali.

To our knowledge, no study has been conducted in Bali examining risk factors for suicide. Although studies from other Asian countries have demonstrated that risk factors for suicide are similar to those in western countries [4-12], culturally or socially unique findings are also noted in each study. For instance, Phillips et al. [6] showed that the presence of a psychiatric disorder was not a significant predictor of suicide in the final regression model in China, although a substantial amount of published work from western countries has reported a link between psychiatric disorders and suicidal behaviors. Moreover, Chen et al. [9] demonstrated that socio-economic adversities such as unemployment and debt have played an important role in suicide in Hong Kong during the recent economic recession. Therefore, in order to establish a culturally appropriate suicide prevention program, it is essential to investigate the risk factors of suicide particular to each region. The psychological autopsy method, which is based on interviews with informants close to the deceased, is currently the most direct technique to examine the relationship between specific factors and suicide [13].

The aim of the present study is to examine socio-demographic, clinical, and psychosocial correlates for suicide in Bali using a case-control psychological autopsy approach. This is the first study to investigate risk factors for suicide in Bali.

## Methods

### Study Area

Bali, located in Southeast Asia, is one of more than 15,000 islands that make up Indonesia. It is famous as a tourist resort and for its unique Hindu-based culture. In 2006, Bali's population was 3,263,296, and the island is almost entirely ethnically and culturally homogeneous. Industry is now in its developing stages on the island. An extended family system is common in Bali, and the basic unit of society is a community referred to as a *banjar*, consisting of up to several hundred households. Most Balinese consider the religious and social activities of the *banjar *to be essential parts of their lives.

### Participants

Sixty-four consecutive suicide cases were extracted from case records for a 4-month period in 2007 from all 53 police districts in Bali. The exact months of death were concealed to protect the identity of each case. The first author (T.K.) collected information regarding the circumstances of death from police, community leaders, doctors, and victims' families. After thoroughly reviewing all relevant information, the research team determined the cause of death to be suicide in all cases. Of the 64 extracted suicide cases, the families of 60 individuals (93.8%) participated in the study. The families of two cases refused to be interviewed. The remaining two families consented to participate in the study; however, they were unable to complete their interviews due to serious emotional distress. These two families were advised to seek appropriate psychiatric help immediately.

For each of the 60 suicide cases, two age- and sex-matched living controls were randomly recruited in the same village. The families of all the controls agreed to participate in the study.

The study was approved by the Ethics Committee of the Indonesian Institute of Science, and all participants gave written informed consent to participate.

### Psychological autopsy interview

For each of the suicide cases and controls, two family members who were most familiar with the individual were chosen as the key informants. Although some subjects lived alone, many relatives lived in the same compound or neighborhood, as an extended family system is common in Bali. The first author (T.K.) conducted face-to-face interviews with all key informants (for both suicide cases and controls) in their homes. Psychological autopsy interviews (for informants of suicide cases) and interviews with informants of controls consisted of the same questions; however, the index date for controls was the date of interview, while it was the date of death for suicide cases. For the informants of suicide cases, the interview was performed between 3 and 12 months (mean 7.3 months; SD 2.82) after the victim's death. Each interview took approximately 2 - 3 hours for families of suicide victims and 1 - 1.5 hours for families of controls.

### Measures

#### Socio-demographic data

The following data were collected: number of family members, marital status, history of divorce, educational status, history of migration, and work status. While many relatives live in the same compound or neighborhood in Bali, only the number of family members living under the same roof was counted in this study.

#### Negative life events

Negative life events that occurred in the 1 year prior to death were assessed using a checklist based on The List of Threatening Experiences [14]. Negative life events were classified into the following eight categories: severe interpersonal problems, severe work-related problems, serious physical illness, severe school-related problems, severe financial problems, serious illness of a family member, death of a close friend or family member, and severe problems with the police. The duration and impact of each negative life event on each individual were recorded.

#### Mental health factors

A structured clinical interview for DSM-IV Axis I disorders (SCID-I) [15] was used to establish the best-estimate current DSM-IV diagnosis over the 1 month prior to death [16]. In this study, axis-I diagnosis included major depressive episode, schizophrenia and other psychotic disorders, substance-related disorders, anxiety disorders, and adjustment disorders. SCID has been translated and back-translated and has proved to be a reliable screening instrument for individuals with schizophrenia in Bali [17]. In this study, for the purpose of accurate diagnosis of depression, we added culturally sensitive terms (e.g., "ngekoh", a frequently used word that means having low energy, no volition, or no will to communicate) to SCID. A similar technique was also employed in a psychological autopsy study in China, as the direct translation of depression is not frequently used in everyday Chinese [6,18]. In addition to psychiatric diagnosis, help-seeking behaviors for psychological problems both over the 1 month prior to death and throughout the individual's lifetime (i.e., seeking psychiatric treatment from mental health professionals, primary health care professionals, or traditional healers) and history of prescriptions for psychiatric medication were also recorded. Medical records were examined to obtain accurate information regarding diagnosis and treatment history whenever available.

#### Psychosocial factors

The following data were collected: previous suicide attempts, family history of suicide, religious activities, and social support.

Religious activities were investigated as follows. Each village in Bali has three temples, and temple anniversary rituals are conducted three times every Balinese calendar year (210 days), each lasting for several days. Participation in these rituals--some of the most important ceremonies conducted in Bali--was used as a standard to gauge individuals' level of religious activity. Individuals who had not participated in any anniversary rituals during the 1 Balinese calendar year prior to death were considered to have a low level of religious activity.

Social support was examined using two modified subscales of the Duke Social Support Index [19]. For the assessment of subjects' size of social network, informants were asked whether the subjects had more than two non-family members in the area (within 1 hour) on whom they could depend or with whom they were very close. For the evaluation of subjects' frequency of social contact, informants were asked whether the subjects spent a significant amount of time with someone who did not live with them more than two times a week, on average, in the 1 month prior to death.

### Coding procedure

Two raters (R.R. and I.T), who were blind to the case-control status, made a psychiatric diagnosis of each subject independently based on the SCID-I interviews. Subjects' negative life events, mental health factors, and psychosocial factors were then coded. After the two raters' psychiatric diagnosis and coding was completed, the first author and the two raters held a consensus meeting to reach a final decision on each item for all subjects, focusing in particular on the items for which the two raters' diagnosis or coding did not coincide and any vague information found in the case records.

### Statistical analysis

Statistical analysis was conducted using SPSS software version 11.5 (SPSS Inc, Chicago, IL, USA). Data were analyzed in a case-control design. First, binary logistic regression analysis was performed to examine the individual effect of each factor on suicide. Odds ratios and their 95% confidence intervals were calculated. Significant variables were then entered into a multivariate logistic regression model, and a back-forward elimination method was employed to identify the most stable model.

## Results

### Description of suicide cases

Of the 60 suicide cases, 38 were males and 22 were females. Mean age was 41.4 (SD 21.5: range 13 - 87) Age distribution was as follows: <15, 2; 15 - 24, 17; 25 - 34, 10; 35 - 44, 5; 45 - 54, 8; 55 - 64, 6; 65 - 74, 6; >74, 6. Forty-one (68.3%) suicide victims died by hanging, 9 (15.0%) by ingestion of poisons, 4 (6.7%) by jumping from a height, 3 (5.0%) by drowning, 2 (3.3%) by stabbing, and 1 (1.7%) by burning.

### Binary logistic regression analysis

Table 1 compares socio-demographic data, negative life events, mental health factors, and psychosocial factors between suicide cases and controls. Only significant variables are listed. Compared with controls, suicide cases were more likely to have a history of divorce, have less education, live alone, and be unemployed.

**Table 1:** Characteristics of suicide cases and controls

	**Suicide cases (n = 60)** **n (percent)**	**Controls (n = 120)** **n (percent)**	**Odds ratio** **(95% CI)**
**Socio-demographic data**			
**History of divorce**	7 (11.7%)	4 (3.3%)	3.83 (1.08-13.65)*
**Low education (≤ 9 years)**	49 (81.7%)	75 (62.5%)	2.67 (1.26-5.67)*
**Living alone**	5 (8.3%)	1 (0.8%)	10.82 (1.23-94.81)*
**Unemployed**	25 (41.7%)	20 (16.7%)	3.57 (1.77-7.21)***
**Negative life events**			
**Severe interpersonal problems**	26(43.3%)	10 (8.3%)	8.41 (3.69-19.19)***
**Severe financial problems**	14 (23.3%)	12 (10.0%)	2.74 (1.18-6.38)*
**Serious physical illness**	14(23.3%)	10 (8.3%)	3.35 (1.39-8.08)**
**Mental health factors**			
**At least one psychiatric diagnosis**	48 (80.0%)	18 (15.0%)	22.67 (10.11-50.80)***
**Past psychiatric treatment**	8 (13.3%)	2 (1.7%)	9.08 (1.86-44.22)**
**Past treatment from traditional healer**	11 (18.3%)	6 (5.0%)	4.27 (1.49-12.18)**
**Psychosocial factors**			
**Low level of religious involvement**	27 (45.0%)	6 (5.0%)	15.55 (5.92-40.83)***
**Small social network**	17 (28.3%)	4 (3.3%)	11.47 (3.65-35.99)***
**Low frequency of social contact**	13 (21.7%)	10 (8.3%)	3.04 (1.25-7.43)*
**Previous suicide attempt**	12 (20.0%)	2 (1.7%)	14.75 (3.18-68.40)**
**Family history of suicide**	10 (16.7%)	3 (2.5%)	7.80 (2.06-29.56)***

*p < 0.05 **p < 0.01 ***p < 0.001

Suicide cases tended to experience more negative life events (i.e., severe interpersonal problems, serious physical illness, and severe financial problems) compared with controls. Suicide victims experienced interpersonal problems with spouses (n = 12; 20.0%), a boy/girlfriend (n = 4; 6.7%), parents (n = 3; 5.0%), children (n = 3; 5.0%), friends (n = 3; 5.0%), and other relatives/persons (n = 8; 13.3%). Seven (26.9%) of 26 suicide cases who experienced severe interpersonal problems had conflicts with multiple persons.

Suicide cases showed a significantly higher prevalence of psychiatric disorders; of 60 suicide cases, 48 (80.0%) had at least one current diagnosis of Axis-I mental disorder. The most prevalent disorder was major depressive episode (n = 31; 51.7%), followed by schizophrenia and other psychotic disorders (n = 9; 15.0%; 6 schizophrenia, 1 schizophreniform disorder, 1 psychotic disorder due to epilepsy, 1 psychotic disorder due to auditory impairment), substance-related disorders (n = 4; 6.7%; 3 alcohol abuse and 1 other substance abuse), adjustment disorders (n = 4; 6.7%), and anxiety disorders (n = 2; 3.3%; social phobia). Of individuals who were diagnosed with mental disorders, two had dual diagnoses; namely, mood disorder co-morbid with substance-related disorder.

Suicide cases were more likely to receive both psychiatric treatment and treatment from traditional healers for psychological reasons in the past than controls. Eight (13.3%) had received psychiatric treatment in their lifetime; however, all had discontinued psychiatric treatment at the time of death. Of 48 suicide cases with mental disorders, 8 (16.7%) had visited a primary health care professional during the 1 month prior to death. Including visits to traditional healers, 12 (25.0%) sought help for their problems (4 at a medical facility, 4 at a traditional healer, and 4 at both) during the 1 month prior to death. However, none of the suicide cases received psychotropic medication.

As for psychosocial factors, suicide cases were more likely to show a low level of religious activity compared with controls. Moreover, suicide victims had a smaller social network and a lower frequency of social contact than controls. Furthermore, suicide cases were more likely than controls to have a history of previous suicide attempts and a family history of suicide.

### Multivariate logistic regression analysis

Multivariate analysis identified three independent significant factors, as shown in Table 2. The presence of at least one psychiatric diagnosis had the strongest effect, followed by low levels of religious involvement and severe interpersonal problems.

**Table 2:** Multivariate model of risk factors for suicide comparing suicide cases and controls

	**Adjusted odds ratio (95% CI)**
**At least one psychiatric diagnosis**	14.84 (6.12 - 35.94)***
**Low level of religious involvement**	7.24 (2.28 - 22.95)**
**Severe interpersonal problems**	3.86 (1.36 - 11.01)*

*p < 0.05 **p < 0.01 ***p < 0.001

## Discussion

In the present study, a wide range of psychosocial and clinical factors that may be related to suicide in Bali were examined. This is the first successful psychological autopsy study in this developing country setting with a high participation rate for both suicide cases and controls, face-to-face direct interviews with key informants, and all informants consisting of close relatives rather than non-family members such as friends and visiting nurses. The results indicated that risk factors for suicide include at least one psychiatric diagnosis, a low level of religious involvement, and severe interpersonal problems.

Mental disorder was the most influential risk factor for suicide in Bali. Robins et al. [20] found that 93% of 109 patients brought to a general hospital immediately after a suicide attempt suffered from psychiatric disorders. According to a systematic review of psychological autopsy studies of suicide [13], mental disorder was the most strongly associated variable of those that had been studied, and the median proportion of cases of mental disorder was 90% (range: 86 - 97%). The results of the present study are therefore consistent with previous psychological autopsy studies, although the percentage of mental disorders in this study was slightly lower (80%) than previously reported. Major depression was the most common diagnosis (51.7%) among suicide cases with mental disorders. This finding is consistent with those from other psychological autopsy studies [13]. On the other hand, alcohol abuse, another predominant mental disorder, was observed in only four suicide cases (6.7%). This finding is not consistent with most other studies except for one study from Pakistan [11], an Islamic country where alcohol is legally banned. Although alcohol is not prohibited by Balinese Hinduism, alcoholism is very rare in Bali, as drinking may cause one to lose his or her sense of geological orientation (e.g., the direction of the holy mountain of Gunung Agung, which represents the positive forces of universe) [21]. Economic factors or the Balinese people's aversion to losing control of their emotions may also contribute to the low prevalence of alcoholism in Bali.

A low level of religious involvement was also found to be a significant risk factor for suicide in Bali. Previous studies in developed countries revealed that religious participation decreases the risk of both suicide completion [22,23] and suicide attempts [24,25]. Possible causes for this effect include the broader social network and increased instrumental support provided by frequent religious participation [24,26]. In Bali, religious ceremonies integrate individuals into the society and provide a forum for the exchange of informational, emotional and tangible social support among community members. In addition, as many religions--including Balinese Hinduism--strongly prohibit suicide, it follows that adherence to these religious doctrines would make followers less likely to attempt suicide [24,26]. Therefore, both social support and religious doctrine appear to contribute to the relationship between religious activities and suicide. The results of the present study showed that religion remained a significant item in the final multiple regression model, while social support items (i.e., size of social network and frequency of social contact) did not; however, it is premature to conclude that religion has a stronger effect on suicide than social support, as the social support items used in this study were not comprehensive. Further examination is needed to explore the relationship between religious involvement and suicide in Bali using more detailed social support criteria. To our knowledge, this is the first psychological autopsy study in an Asian developing country to indicate a significant association between religious activities and suicide completion.

Severe interpersonal problems were also identified as a risk factor for suicide in the present study. This finding is similar to those from Western countries [27-29] and Asian countries [6,8,10]. Interpersonal problems were more directly related to suicide than other negative life events, such as financial problems, in this study. In general, Balinese live in communities with high population densities where frequent community activities create complicated interactions among families and community members. In such a social setting, severe interpersonal problems may reach a critical point and make an individual more likely to attempt suicide.

The present study revealed several important implications for suicide prevention in Bali. The strongest risk factor for suicide identified in the present study was mental disorder--48 (80.0%) suicide cases were diagnosed with mental disorders. However, only 16.7% of these cases contacted a primary health care professional, and none had received any psychiatric treatment during the 1 month prior to death. This finding is contrary to results from developed countries, where a majority of suicide cases had visited a primary care physician during the 1 month before death [30,31]. In Bali, public education aimed at fostering help-seeking behaviors in individuals with mental disorders is essential for suicide prevention. In addition, education programs for primary care health professionals on the recognition and management of mental disorders are important, and the accessibility and availability of mental health care in Bali must be improved. However, Chen and Yip [32] point out that increasing the number of psychiatric professionals may not directly translate into a decrease in suicide rate, as the suicide rate in Taiwan has increased three-fold in the past decade despite a 100% increase in the number of psychiatrists. They argue that a community-based method, rather than a psychiatric or clinical approach, is more relevant and cost-effective in Asian countries. Although the treatment of mental disorders in Bali is essential, such community-based prevention strategies also appear to be important. In the present study, a low level of religious activities and severe interpersonal relationship problems were also identified as significant risk factors. For the former, community-based intervention by religious leaders may be beneficial, while for the latter, consultation with either community leaders or traditional healers may be effective. For instance, religious leaders may be able to inquire about the absence of withdrawn individuals from religious ceremonies and offer the individuals or their families a religious solution, and community leaders could function as mediators of interpersonal conflicts within the community. Through those processes, religious and community leaders may be able to develop and reinforce social networks to support individuals at risk of committing suicide.

The present study has several limitations. First, the methodological problems inherent to psychological autopsy studies should be noted--the interviewer was not blind to suicide/control status, and the long duration between time of death and interview may have caused recall bias on the part of the informants. However, in the present study, a careful consensus meeting was held for the coding process of each factor in an attempt to minimize bias. Second, the present study's small sample size makes the interpretation of the results rather difficult. Third, data extracted from police reports may be a major source of bias due to the underreporting of suicides.

## Conclusion

In summary, the present study identified at least one psychiatric diagnosis, a low level of religious involvement, and severe interpersonal problems as risk factors for suicide in Bali. Both clinical and community-based suicide prevention strategies are essential. Future studies should evaluate which aspects of prevention strategies are effective for establishing the most relevant suicide prevention program in Bali.

## Competing interests

The authors declare that they have no competing interests.

## Authors' contributions

TK drafted the study design, carried out data collection, participated in interpretation of the data, performed statistical analysis, and drafted the manuscript. MK provided advice on the study design, performed statistical analysis, and reviewed the manuscript. RR and IT participated in interpretation of the data and reviewed the manuscript. All authors read and approved the final manuscript.

## Pre-publication history

The pre-publication history for this paper can be accessed here:


